# CROSSLINKER CONCENTRATION CONTROLS TGFβ−3 RELEASE AND ANNULUS FIBROSUS CELL APOPTOSIS IN GENIPIN-CROSSLINKED FIBRIN HYDROGELS

**DOI:** 10.22203/eCM.v039a14

**Published:** 2020-05-12

**Authors:** C.J. Panebianco, T.J. DiStefano, B. Mui, W.W. Hom, J.C. Iatridis

**Affiliations:** Leni and Peter W. May Department of Orthopaedics, Icahn School of Medicine at Mount Sinai, New York, NY, USA

**Keywords:** Intervertebral disc, annulus fibrosus repair, injectable biomaterials, hydrogel, cell delivery, growth factor delivery, fibrin, genipin crosslinking, apoptosis, musculoskeletal tissue engineering

## Abstract

Back pain is a leading cause of global disability associated with intervertebral disc (IVD) pathologies. Discectomy alleviates disabling pain caused by IVD herniation without repairing annulus fibrosus (AF) defects, which can cause accelerated degeneration and recurrent pain. Biological therapies show promise for IVD repair but developing high-modulus biomaterials capable of providing biomechanical stabilisation and delivering biologics remains an unmet challenge. The present study identified critical factors and developed an optimal formulation to enhance the delivery of AF cells and transforming growth factor beta-3 (TGFβ−3) in genipin-crosslinked fibrin (FibGen) hydrogels. Part 1 showed that AF cells encapsulated in TGFβ−3-supplemented high-modulus FibGen synthesised little extracellular matrix (ECM) but could release TGFβ−3 at physiologically relevant levels. Part 2 showed that AF cells underwent apoptosis when encapsulated in FibGen, even after reducing fibrin concentration from 70 to 5 mg/mL. Mechanistic experiments, modifying genipin concentration and integrin binding site presence demonstrated that genipin crosslinking caused AF cell apoptosis by inhibiting cell-biomaterial binding. Adding integrin binding sites with fibronectin partially rescued apoptosis, indicating genipin also caused acute cytotoxicity. Part 3 showed that FibGen formulations with 1 mg/mL genipin had enhanced ECM synthesis when supplemented with fibronectin and TGFβ−3. In conclusion, FibGen could be used for delivering biologically active compounds and AF cells, provided that formulations supplied additional sites for cell-biomaterial binding and genipin concentrations were low. Results also highlighted a need for developing strategies that protect cells against acute crosslinker cytotoxicity to overcome challenges of engineering high-modulus cell carriers for musculoskeletal tissues that experience high mechanical demands.

## Introduction

Back pain and associated radicular pain are leading causes of global disability, accounting for approximately $85.9 billion in healthcare spending in the United States (Hartvigsen *et al.*, 2018; Martin *et al.*, 2008). There is a strong association between back pain and herniation, degeneration and modic changes of the IVD (Adams and Dolan, 2012; Livshits *et al.*, 2011; Mok *et al.*, 2016). The most common procedure performed to alleviate disabling leg and back pain resulting from IVD herniation is discectomy, whereby herniated NP tissue is removed to alleviate pressure against the spinal nerve column. While effective at reducing pain from neuropathy (Asch *et al.*, 2002; Gray *et al.*, 2006; Lurie *et al.*, 2014; Weinstein *et al.*, 2008), discectomy does not repair AF defects created by NP herniation, causing 10–30 % of patients to experience further disc degeneration, reherniation and recurrent pain (Abdu *et al.*, 2017; Carragee *et al.*, 2003; McGirt *et al.*, 2009; Watters and McGirt, 2009). To prevent these types of complications, there is a critical need to develop AF repair strategies that restore IVD function and prevent recurrent pain.

A simple solution for repairing AF defects is suturing; however, commercially available suturing systems, such as Xclose™ (Anulex Technologies, Minnetoka, MN, USA; Bailey *et al.*, 2014), do not significantly reduce reherniation risks and have been removed from the United States market (Bailey *et al.*, 2013). More complex implantable AF closure devices, such as Barricaid® (Intrinsic Therapeutics, Woburn, MA, USA; Parker *et al.*, 2016), showed promise for preventing reherniation (Lequin *et al.*, 2012; Parker *et al.*, 2016; Wilke *et al.*, 2013) but do not promote tissue regeneration or demonstrate a capacity to restore biomechanical function. Tissue engineering offers potential for regenerative AF repair because experimental biomaterials can replicate IVD properties and promote cell-mediated remodelling of IVD tissue (Bowles and Setton, 2017). Ideal biomaterial candidates for AF repair should be injectable, to allow easy administration, restore disc height, withstand regular multidimensional loading on the spine and degrade slowly while promoting long-term healing (Guterl *et al.*, 2013; Iatridis *et al.*, 2013; Long *et al.*, 2016). FibGen is an injectable hydrogel that meets many of these design criteria. Moreover, after injury, acellular FibGen injection partially restores IVD height loss and biomechanical function with little herniation risk (Likhitpanichkul *et al.*, 2014; Schek *et al.*, 2011).

While high-modulus biomaterials, which mimic the material properties of the AF tissue, are promising for sealing AF defects, they cannot intrinsically promote healing. Alternatively, cellular therapies using IVD cells (Sakai and Andersson, 2015; Schol and Sakai, 2019) and growth factor delivery (Evans, 2006) have shown promise for slowing IVD degeneration and reducing pain in both preclinical models and clinical trials. Consequently, the study hypothesis was that delivering biological factors in a high-modulus carrier could provide immediate biomechanical stability and promote long-term healing. Previous studies of FibGen demonstrated that encapsulated bovine AF cells maintain high viability *in vitro*, suggesting that FibGen could be a promising cell carrier. However, encapsulated AF cells in the most biomechanically favourable FibGen formulations synthesised the least amount of ECM proteins (Cruz *et al.*, 2018).

Maintaining the balance between mechanical performance and biological function is an ubiquitous challenge when attempting to repair musculoskeletal soft tissues, such as IVD (D’Este *et al.*, 2018) and tendon (Yan *et al.*, 2018), that experience high mechanical demands. Low-modulus cell delivery biomaterials, which have materials properties significantly inferior to those of the AF tissue, including alginate (Guillaume *et al.*, 2014), gelatine (Wan *et al.*, 2008), collagen (Borde *et al.*, 2015) and fibrin (Colombini *et al.*, 2014), do promote cell function; however, these materials do not match IVD biomechanical properties and cannot provide early mechanical stabilisation under high-magnitude spinal loading. Cytocompatible crosslinkers can be used to improve the biomechanical properties of these biomaterials (Oryan *et al.*, 2018) but encapsulated cells have hampered performance (D’ Este *et al.*, 2018) and there are relatively few studies mechanistically investigating why cells perform poorly in highly crosslinked, high-modulus biomaterials. Improving the design of next-generation cell delivery biomaterials for IVD tissue engineering requires more research to better understand how cells interact with macromers and crosslinkers of high-modulus biomaterials.

The purpose of the present study was to track the fate of encapsulated cells and growth factors in high-modulus FibGen in order to establish the critical design criteria requiring optimisation. The present three-part, cell culture study systematically varied FibGen formulations and evaluated their interactions with AF cells and TGFβ−3 growth factor. Bovine AF cells, isolated from caudal IVDs, were used because they are readily available and easily translatable to a large animal model that experiences similar nutrition and loading to human IVDs (Alini *et al.*, 2008). FibGen was used as the candidate biomaterial because it is an adhesive, high-modulus biomaterial that matches AF shear properties and exhibits low herniation risk (Likhitpanichkul *et al.*, 2014). Part 1 was an ECM synthesis and TGFβ−3 release study that determined whether dynamic culture of high-modulus cell-laden FibGen formulations could stimulate substantial ECM synthesis and quantified the TGFβ−3 release kinetics from these constructs. TGFβ−3 supplementation and dynamic culture have been shown to increase AF cell ECM synthesis *in vitro* (Abbott *et al.*, 2013; Agrawal *et al.*, 2018; Hondke *et al.*, 2018; Nerurkar *et al.*, 2011). Part 2a was a FibGen macromer titration study that determined the role of fibrin macromer concentration on encapsulated AF cell apoptosis levels. The macromer concentration range spanned from high (material properties similar to native AF tissue) to low modulus (material properties inferior to those of native AF tissue). Apoptosis was investigated as an output measurement to determine if it was the reason for limited ECM synthesis. Part 2b was a FibGen crosslinker mechanistic study that determined if genipin crosslinking induced encapsulated cells to undergo apoptosis and if modified integrin binding sites was the underlying mechanism. Part 3 was a fibronectin and TGFβ−3 supplementation experiment that characterised the contributions of fibronectin and TGFβ−3 to ECM synthesis and TGFβ−3 release in the formulation from part 2 that resulted in the least amount of apoptosis.

## Materials and Methods

### Study design

**Fig. 1.**
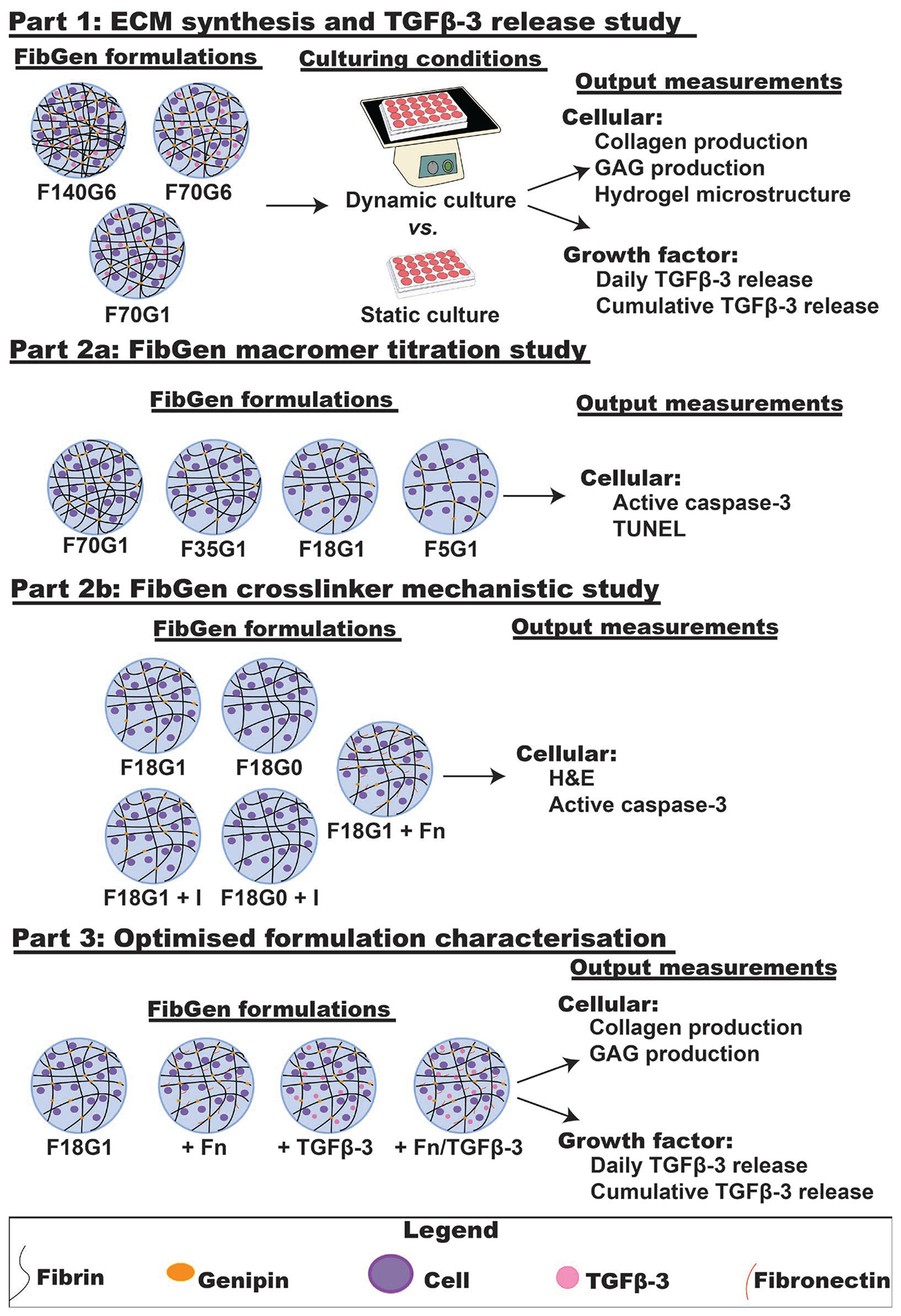
Schematic diagram of methods and outcome measurements for the three-part study carried out. Part 1 was an ECM synthesis TGFβ−3 release study that determined whether dynamic culture of high-modulus cell-laden FibGen formulations could stimulate substantial ECM synthesis and quantified the TGFβ−3 release kinetics from these constructs. Part 2a was a FibGen macromer titration study, which determined the role of fibrin macromer concentration on encapsulated AF cell apoptosis levels using a wide range of FibGen formulations. Part 2b was a FibGen crosslinker mechanistic study that determined if genipin crosslinking induced encapsulated cells to undergo apoptosis and if the lack of cell-biomaterial binding was the mechanism. Part 3 was an optimised formulation characterisation study that assessed ECM synthesis and TGFβ−3 release for formulations that reduced apoptosis in part 2. FibGen formulation abbreviations denote final concentrations of fibrin and genipin used (*e.g*. F140G6 contained 140 mg/mL fibrin and 6 mg/mL genipin). Fibrin, genipin and fibronectin molecules are represented by black lines, orange circles and red lines, respectively. Encapsulated AF cells and TGFβ−3 are represented by purple and pink circles, respectively.

The project included a three-part study design (Fig. 1). Part 1 was a 16 d ECM synthesis and TGFβ−3 release study that used histological staining and SEM to measure ECM synthesis and ELISA to quantify TGFβ−3 release kinetics in multiple high-modulus FibGen formulations. Part 2a was a 7 d FibGen macromer titration study that used immunohistochemical staining and TUNEL to evaluate apoptosis levels in reduced macromer-concentration FibGen formulations. Part 2b was a 5 d FibGen crosslinker mechanistic study that used histological staining to assess cell-biomaterial binding and apoptosis levels in FibGen with and without genipin and with either increased or decreased integrin binding sites using fibronectin or an inhibitor of integrin recognition sites. Part 3 was a 16 d study characterising the contributions of fibronectin and TGFβ−3 to ECM synthesis and TGFβ−3 release in the formulation from part 2 that resulted in the least amount of apoptosis. Histological staining and ELISA were used to measure ECM synthesis and TGFβ−3 release kinetics, respectively.

### Cell isolation and expansion

Skeletally mature bovine tails (*N* = 3 animals) were collected from local abattoirs (Green Village Packing Co., Green Village, NJ, USA and Springfield Meat Co., Richlandtown, PA, USA) and processed independently within 4 h of sacrifice. Skin, fat and muscle tissues were removed to expose caudal IVDs, which were subsequently dissected from adjacent vertebral bodies and placed in 1× PBS (Fisher Scientific™). IVDs were washed with 70 % ethanol, followed by a wash solution of 1.5 % Fungizone (Fisher Scientific™) and 3 % PS (Fisher Scientific™) in 1× PBS under sterile conditions. The AF was isolated from the NP, cut into small (~ 3 mm^3^) pieces, sterilely transferred to T75 Nunc™ EasYFlask™ cell culture flasks (Fisher Scientific™) with the addition of 25 mL of 0.2 % pronase (Fisher Scientific™) solution dissolved in DMEM (Fisher Scientific™) and incubated for 90 min at 37 °C and 20 % O_2_ on a shaker in a humidified incubator (Napco Series 8000 WJ; Thermo Fisher Scientific). Partially digested AF tissue was washed twice with 1× PBS to remove pronase, then digested using 200 U/mL collagenase I (Fisher Scientific™) dissolved in DMEM for 13–17 h at 37 °C and 20 % O_2_ on a shaker in a humidified incubator. Digested AF tissue was filtered through a 70 μm filter (Fisher Scientific™), centrifuged in an Eppendorf® centrifuge 5702 (Sigma-Aldrich) at 500 ×*g* for 10 min and the resulting cell pellet was counted using the Invitrogen Countess automated cell counter (Fisher Scientific™). AF cells were seeded at a density of 4.4 × 10^3^ cells/cm^2^ and expanded in high-glucose DMEM (Fisher Scientific™) supplemented with 10 % FBS (Gemini Bio-Products, West Sacramento, CA, USA), 1 % PS and 0.2 % ascorbic acid (Fisher Scientific™) in a humidified incubator at 37 °C and 20 % O_2_. Medium was changed every 2–3 d and cultures were passaged at 90–95 % confluence using TrypLE™ Express Enzyme (Fisher Scientific™).

### Hydrogel fabrication and culture

FibGen formulations were mixed using a 4 : 1 dual-barrel syringe with mixing tip (Pacific Dental, Walnut, CA, USA). The large syringe barrel contained fibrinogen (Sigma-Aldrich) dissolved in 1× PBS that was mixed thoroughly with DMEM containing bovine AF cells at 20 M cells/mL and 700 ng/mL TGFβ−3 (R&D Systems). The small syringe barrel contained serum-free DMEM, 28 U/mL thrombin (Sigma-Aldrich) and genipin (Sigma-Aldrich) dissolved in DMSO (Sigma-Aldrich). After mixing, FibGen was extruded from the 4 : 1 dual barrel syringe with mixing tip into 5 × 5 mm cylindrical moulds and placed in a humidified incubator for 3–4 h to allow for polymerisation and crosslinking to occur. FibGen formulation abbreviations denote final concentrations of fibrin and genipin used in each part of the study (*e.g*. F140G6 contains 140 mg/mL fibrin and 6 mg/mL genipin). F140G6, F70G6 and F70G1 formulations were noted as high-modulus FibGen formulations because they mimicked the Young’s compressive and complex shear moduli of human AF tissue (Cruz *et al.*, 2018). Remaining formulations with reduced macromer and crosslinker concentrations were noted as low modulus.

TGFβ−3 was not included in the large syringe barrel for part 2. Genipin was not included in the small syringe barrel for F18G0 formulations in part 2b. 10 μg/mL bovine fibronectin (Sigma-Aldrich) and 1.24 mg/mL human factor XIII (Sigma-Aldrich) were included in the large syringe barrel, with 10 mM CaCl_2_ (Sigma-Aldrich) in the small syringe barrel, for the F18G1 + Fn formulation in parts 2b and 3, to add integrin binding sites.

For part 1, AF cells from one animal (*N* = 1 biological replicate) were seeded into previously published hydrogel formulations (Cruz *et al.*, 2018), with 3 technical replicates (*n* = 3) per formulation, per output measurement. For parts 2 and 3, AF cells from 3 animals (*N* = 3 biological replicates) were seeded into experimental hydrogel formulations with 3 technical replicates (*n* = 3) per output measurement (Fig. 1). Genipin concentration was held constant at 1 mg/mL for part 2a because previously published work showed that AF cells seeded in FibGen hydrogels with 1 mg/mL genipin display the highest viability (Cruz *et al.*, 2018). A constant fibrin concentration of 18 mg/mL was chosen for all FibGen formulations in parts 2b and 3 because similar density hydrogels have been shown to support cell viability without rapid degradation (Breen *et al.*, 2009).

FibGen hydrogel constructs were cultured in Corning™ 24-well cell culture plates (Fisher Scientific™) with expansion medium for 16, 7, 5 and 16 d for parts 1, 2a, 2b and 3, respectively. Tirofiban hydrochloride (Selleckchem, Houston, TX, USA), an inhibitor of the αIIbβ3 integrin recognition site on fibrin, was included in the medium of + I conditions of part 2b at a concentration of 30 nM to prevent AF cells from binding to the fibrin matrix. Unless otherwise stated, FibGen constructs were cultured dynamically, meaning constructs were continuously cultured on a BenchRocker™ 2D Rocker (Alkali Scientific Inc., Fort Lauderdale, FL, USA) at the highest rocking setting.

### ECM synthesis

#### Collagen I IHC

Chromogenic IHC was used to assess the synthesis of collagen I (parts 1 and 3). Cell-laden hydrogels cultured for 16 d were fixed in aqueous-buffered zinc formalin fixative (Anatech Ltd., Battle Creek, MI, USA). Samples were fixed for 24 h, processed and paraffin-wax-embedded using the Leica HistoCore Arcadia H and C. A Leica RM 2165 Rotary Microtome was used to create 5 μm-thick sections. Sections were mounted on Millennia™ 1000 Adhesion Slides (StatLab Medical Products, McKinney, TX, USA) and slides were incubated overnight in a dry incubator at 60 °C. Slides were rinsed in petroleum ether (Fisher Chemical, Fair Lawn, NJ, USA) and ethylene glycol monomethyl ether (Fisher Chemical) solutions to dewax the sections. After dewaxing, slides were washed three times with DI water and incubated with Dako Protein Block, Serum-Free for 30 min. After blocking, Rabbit Anti-Collagen I primary antibody (ab34710; Abcam) diluted 1 : 150 in Dako Antibody Diluent, Background Reducing was applied and sections were incubated overnight at 4 °C in a humidity chamber. After overnight incubation, slides were dipped in 1× PBS-Tween® 20 (Pierce Biotechnology, Rockford, IL, USA) three times and washed thrice with DI water. ImmPRESS® Goat Anti-Rabbit IgG Polymer Detection Kit, Peroxidase secondary antibody (Vector Laboratories) was applied and slides were incubated for 30 min at room temperature in a humidity chamber. After secondary antibody incubation, slides were dipped three times in 1× PBS-Tween® 20 and washed three times with DI water prior to 10 min incubation in 3 % H_2_O_2_ (Fisher Chemical). Then, slides were washed thrice with DI water and incubated with ImmPACT® DAB Substrate, Peroxidase (HRP) (Vector Laboratories) for 60 s at room temperature. Following chromogen incubation, slides were dipped three times in 1× PBS-Tween® 20 and washed three times with DI water. After staining, samples were counterstained with toluidine blue O solution (Sigma-Aldrich) for 2 min at room temperature. Counterstained samples were washed thrice with DI water and dipped in solutions of ethylene glycol monomethyl ether and xylene (Fisher Chemical) for dehydration. After dehydration, EUKITT® Mounting Medium (Electron Microscopy Sciences, Hatfield, PA, USA) was used to apply Fisherfinest™ Premium Cover Glass (Fisher Scientific™) to slides. Brightfield microscopy using a Zeiss Axio Imager Z1 was used to visualise the sections. Collagen I immunopositivity was quantified using 20× objective-lense-images of three regions of interest per hydrogel. 63× objective-lense-images were used for the figures to highlight immunopositive and immunonegative cells.

The anti-collagen I antibody, which has species reactivity towards bovine and mouse, was validated using the mouse IVD as an antigen-positive tissue control. Negative control rabbit IgG (Biocare Medical) was used as an isotype control.

#### Tinctorial staining

Picrosirius red (Poly Scientific R&D Group, Bay Shore, NY, USA) and alcian blue (Poly Scientific R&D Group) tinctorial stainings were used to assess collagen and GAG content, respectively. Slides were dewaxed, rinsed three times in DI water and stained with picrosirius red solution or alcian blue for 1 h or 30 min, respectively. Following staining, slides were rinsed in 1 % acid water for 2 min, then dehydrated and cover-slipped before images were acquired by brightfield microscopy.

### Growth factor release kinetics

Cell culture medium was collected every other day, prior to media exchange, from statically and dynamically cultured FibGen constructs, for analysis by solid phase sandwich ELISA (Human TGF-beta 3 DuoSet ELISA, R&D Systems). Active human TGFβ−3 concentration was measured over time in cell culture medium as per manufacturer’s instructions. The optical density of each well was measured at 450 nm using a microplate reader (Molecular Devices SpectraMax i3x, San Jose, CA, USA). All experimental measurements were performed in triplicate. Statically and dynamically cultured constructs were pooled for analysis because no statistically significant differences were found based on culturing method.

### Hydrogel microstructure

FibGen microstructure was visually assessed by SEM. Cell-laden FibGen hydrogels were fixed using 3 % glutaraldehyde, then 1 % osmium tetroxide prior to dehydration using ethyl alcohol. Dehydrated samples were gold-sputtered for SEM using an S4300 SEM at 5 kV (Hitachi High Technologies, Ibaraki, Japan).

### Apoptosis detection

Apoptosis levels of cells in different hydrogel formulations were assessed using chromogenic IHC for active caspase-3 (Collagen IHC paragraph) and TUNEL. For IHC, rabbit polyclonal Anti-Cleaved Caspase-3 antibody (ab49822; Abcam) was used at a dilution of 1 : 5,000. Immunostaining was validated by processing the mouse growth plate as an antigen positive/negative tissue control and a negative isotype control (Collagen IHC paragraph). For TUNEL, the Click-iT™ Plus TUNEL Assay for In Situ Apoptosis Detection, Alexa Fluor™ 488 dye (Thermo Fisher Scientific) was used according to the manufacturer’s instructions. TUNEL staining was validated by processing positive controls, treated with DNAse I, Amplification Grade Kit according to Manufacturers Instructions (Fisher Scientific™) for 30 min to induce DNA breaks, and negative controls, processed without the TdT enzyme to ensure the TdT reaction would not proceed alongside experimental sections. Active caspase-3 and TUNEL positivity were quantified using 20× objective-lense-images images of three regions of interest per hydrogel. 63× objective-lense-images images were used for the figures to highlight positive and negative cells stained with active caspase-3 or nuclei co-stained with DAPI and TUNEL.

### Cellular morphology

Morphology of FibGen-encapsulated AF cells was visually assessed by H&E staining. Slides were dewaxed and stained using haematoxylin solution (Fisher Scientific™) for 10 min. Following staining, slides were rinsed in DI water three times and incubated in eosin Y solution (Fisher Scientific™) for 2 min. After staining, sections were dehydrated and cover-slipped before images were acquired by brightfield microscopy.

### Statistical analysis

GraphPad Prism software Version 7 (Prism7) was used to conduct all statistical analysis, with results represented as mean ± standard deviation. Two-way ANOVA with Bonferroni correction for multiple comparisons at α = 0.05 was used to determine significant differences in collagen I (part 1) and apoptosis marker (part 2a) positivity between groups. One-way ANOVA with Tukey’s *post-hoc* test at α = 0.05 was used to determine significant differences in TGFβ−3 release (parts 1 and 3), active caspase-3 immunopositivity (part 2b) and collagen I immunopositivity (part 3) between groups.

## Results

### ECM synthesis study

**Fig. 2.**
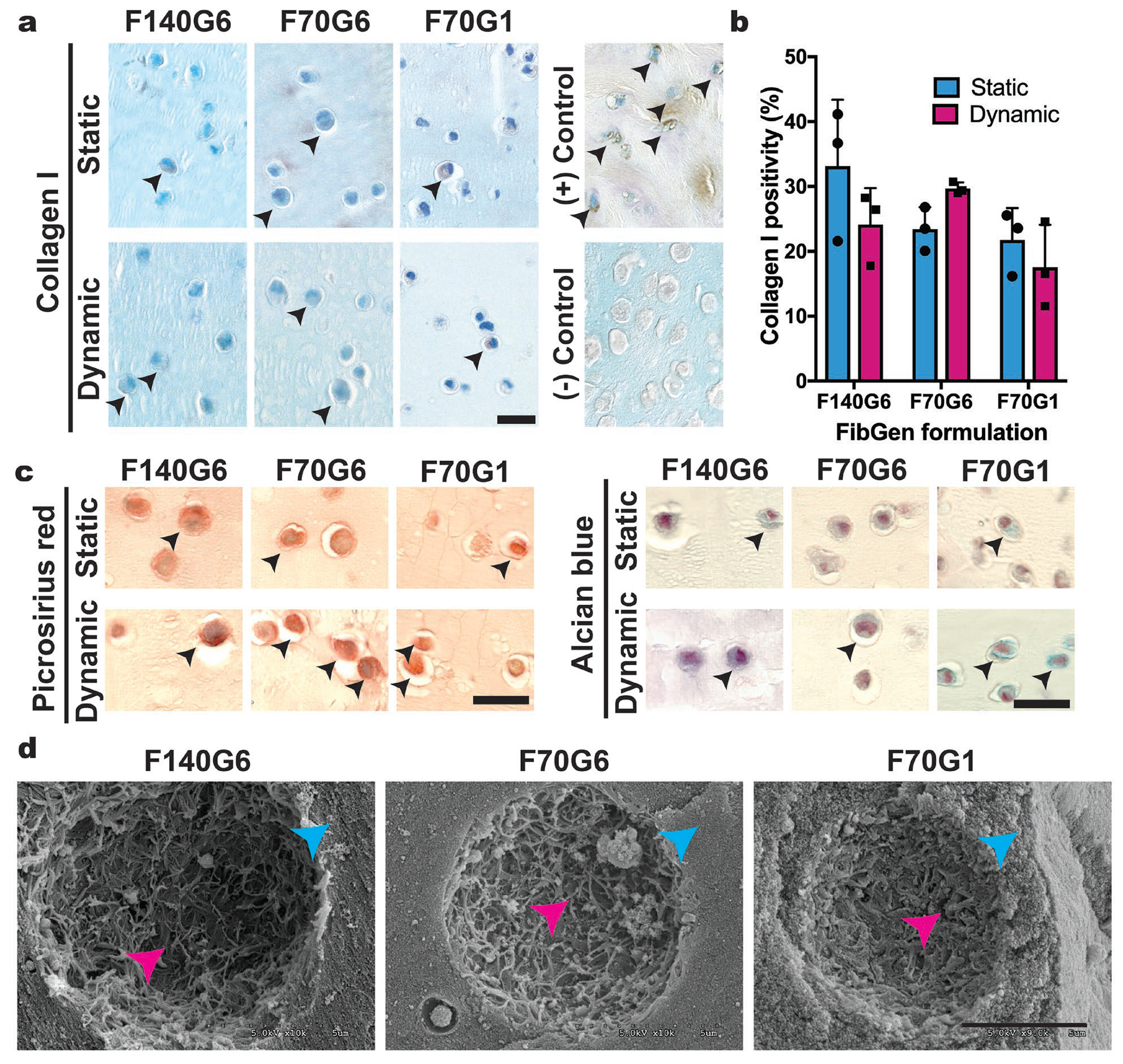
Dynamic culture with TGFβ−3 did not promote significant matrix elaboration. (**a**) Day 16 collagen I immunostaining of high-modulus FibGen formulations. Black arrowheads indicate collagen I positive cells. Scale bar: 20 μm. (**b**) Semiquantitative analysis of collagen I positivity indicated no significant differences between groups or culturing conditions. Data represented as mean ± standard deviation. (**c**) Day 16 picrosirius red and alcian blue tinctorial staining of high-modulus FibGen formulations. Black arrowheads indicate positive staining for collagen content and proteoglycan/GAG deposition in the pericellular space. Scale bar: 20 μm. (**d**) SEM images showed extremely dense FibGen hydrogel network with ECM synthesis confined to the pericellular space. Pink and cyan arrowheads indicate surface topography of the fibrillar network with concave pericellular spaces and the surrounding FibGen gel, respectively. Scale bar: 5 μm. *N* = 1 biological replicate; *n* = 3 technical replicates.

To evaluate the capacity of FibGen-encapsulated bovine AF cells to synthesise ECM under static and dynamic culturing conditions with TGFβ−3 supplementation, hydrogels cultured for 16 d were stained for collagen I and with picrosirius red and alcian blue. Three high-modulus FibGen formulations were assessed: F140G6, F70G6 and F70G1, where FibGen formulation abbreviations denote final concentrations of fibrin and genipin used (*e.g*. F140G6 contains 140 mg/mL fibrin and 6 mg/mL genipin). Semiquantitative analysis of collagen I IHC (Fig. 2a) showed no significant differences in collagen I immunopositivity based on FibGen formulation or culturing conditions (Fig. 2b). This was confirmed by picrosirius red and alcian blue tinctorial stainings, which showed limited positivity for collagen and GAGs in all formulations (Fig. 2c). Qualitative assessment of tinctorial staining demonstrated slightly more ECM synthesis in dynamically cultured FibGen constructs as compared to statically cultured controls. Picrosirius red staining qualitatively showed slightly higher positivity than collagen I IHC since it stains all collagen types and AF cells phenotypically synthesise numerous types of collagen (van den Akker *et al.*, 2017). SEM imaging showed that ECM synthesis was limited to the pericellular space in all tested FibGen formulations (Fig. 2d).

### TGFβ−3 release study

**Fig. 3.**
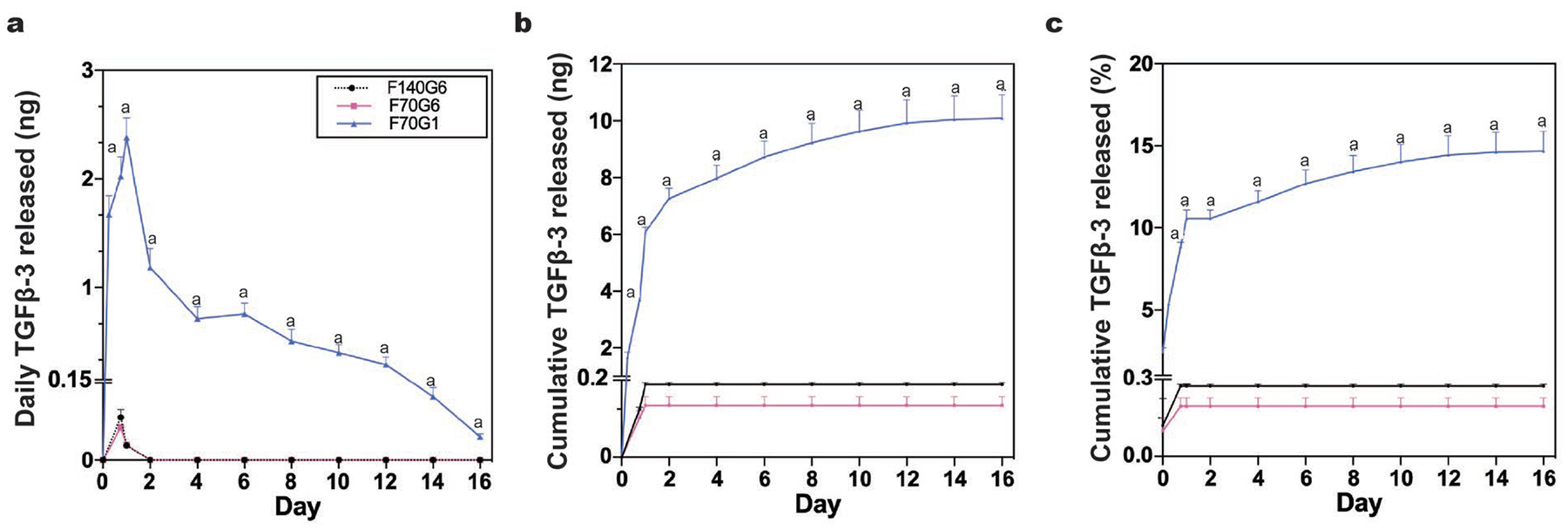
Genipin crosslinker concentration significantly affected TGFβ−3 release kinetics. The F70G1 formulation had significantly increased daily and cumulative release of TGFβ−3 when compared to the F140G6 and F70G6 groups (^a^ *p* < 0.0001). Data represented as mean ± standard deviation.

ELISA analysis of media used to culture TGFβ−3-loaded FibGen hydrogels showed that dense FibGen formulations were capable of growth factor release; however, FibGen formulations containing 1 mg/mL genipin released significantly more TGFβ−3 than formulations containing 6 mg/mL genipin (Fig. 3). All formulations exhibited the largest TGFβ−3 release within the first 48 h of culture, followed by tapered daily release from day 2 onwards (Fig. 3a). Medium concentrations of TGFβ−3 during this burst release were 1.18 ± 1.02 pM, 0.96 ± 0.88 pM and 28.5 ± 4.31 pM for F140G6, F70G6 and F70G1 formulations, respectively. At culture day 16, the medium concentration of TGFβ−3 was reduced to 0.64 ± 0.15 pM for the F70G1 formulation and undetectable for the F140G6 and F70G6 formulations. Cumulative growth factor release showed a steady increase over the 16 d culture period for the F70G1 formulation, resulting in 14.68 ± 1.47 % release of total loaded TGFβ−3. F140G6 and F70G6 formulations displayed negligible cumulative TGFβ−3 release after culture day 2, releasing 0.18 ± 0.11 % and 0.13 ± 0.08 % of the loaded TGFβ−3, respectively (Fig. 3c).

### FibGen macromer titration study

**Fig. 4.**
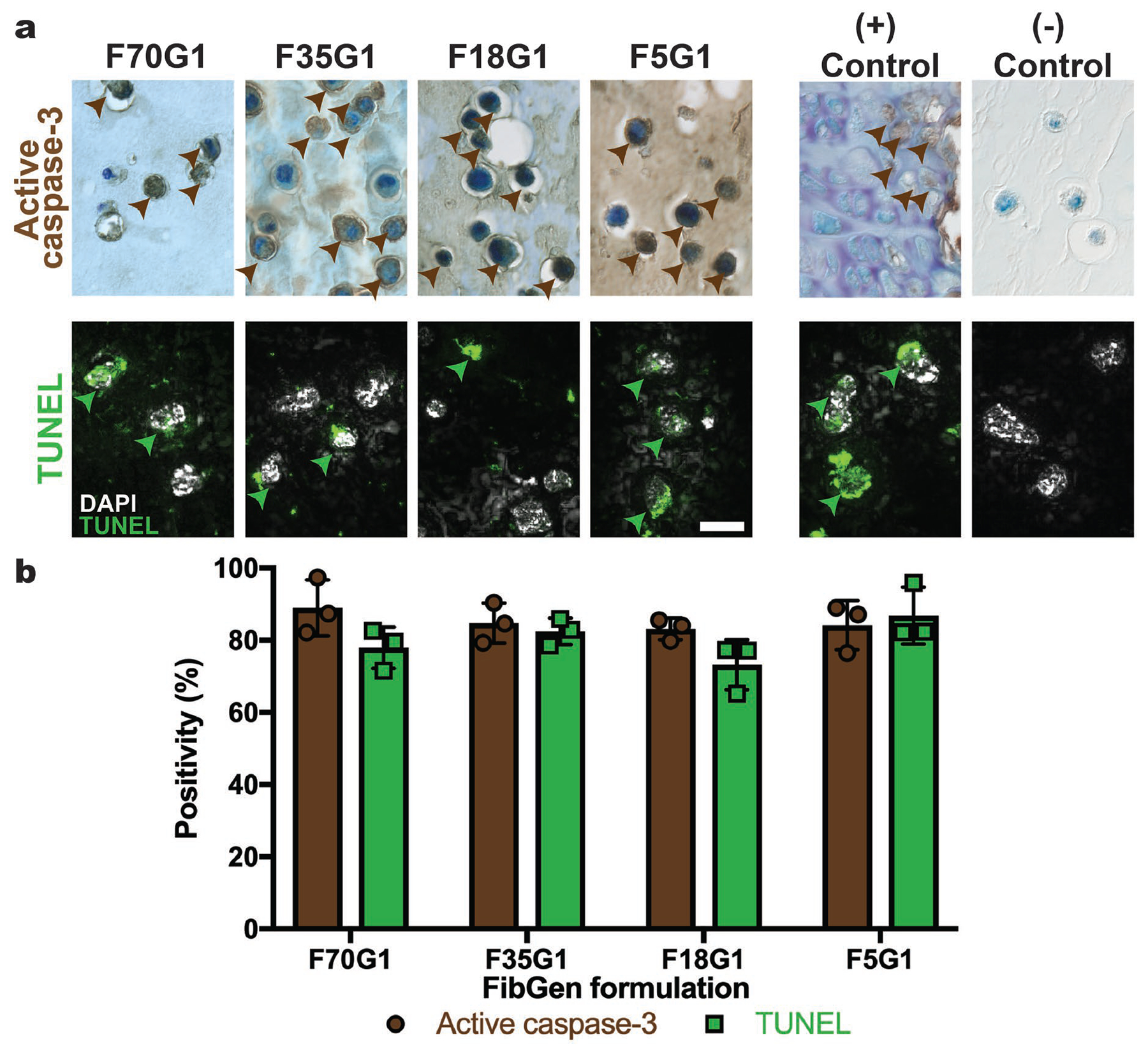
All FibGen formulations containing 1 mg/mL genipin had apoptosis levels higher than 75 %. (**a**) Day 7 active caspase-3 and TUNEL staining of FibGen formulations with decreased fibrin macromer concentration. Brown and green arrowheads indicate apoptotic cells. Scale bar: 20 μm. (**b**) Semiquantitative analysis of active caspase-3 and TUNEL positivity indicated no significant differences in apoptosis levels between groups or apoptosis detection method. Data represented as mean ± standard deviation. *N* = 3 biological replicates; *n* = 3 technical replicates per biological replicate.

Visual analysis of dense FibGen hydrogel surface topography using SEM revealed an extremely dense hydrogel network with no distinguishable pores on the micron scale. This structure confined cells to rounded pockets that limited ECM synthesis to the pericellular space (Fig. 2d). This observation prompted an investigation of the effect of fibrin macromer concentration on encapsulated cell health. Fibrin concentration was reduced from 70 mg/mL (lowest concentration used in part 1) to 5 mg/mL. Reducing fibrin macromer concentration and conducting semiquantitative analysis of apoptosis levels, using active caspase-3 IHC and TUNEL (Fig. 4a), showed that FibGen encapsulation caused high levels of apoptosis (Fig. 4b). Furthermore, two-way ANOVA analysis revealed no significant differences in apoptosis levels between FibGen formulations or apoptosis detection method. The high apoptosis levels in the F70G1 formulation indicated that the low ECM synthesis in part 1 was related to cellular apoptosis. Furthermore, the high apoptosis levels in FibGen formulations with lower fibrin macromer concentrations indicated that the genipin concentration of 1 mg/mL, and not the high fibrin macromer concentration, caused encapsulated AF cells to undergo apoptosis.

### FibGen crosslinker mechanistic study

**Fig. 5.**
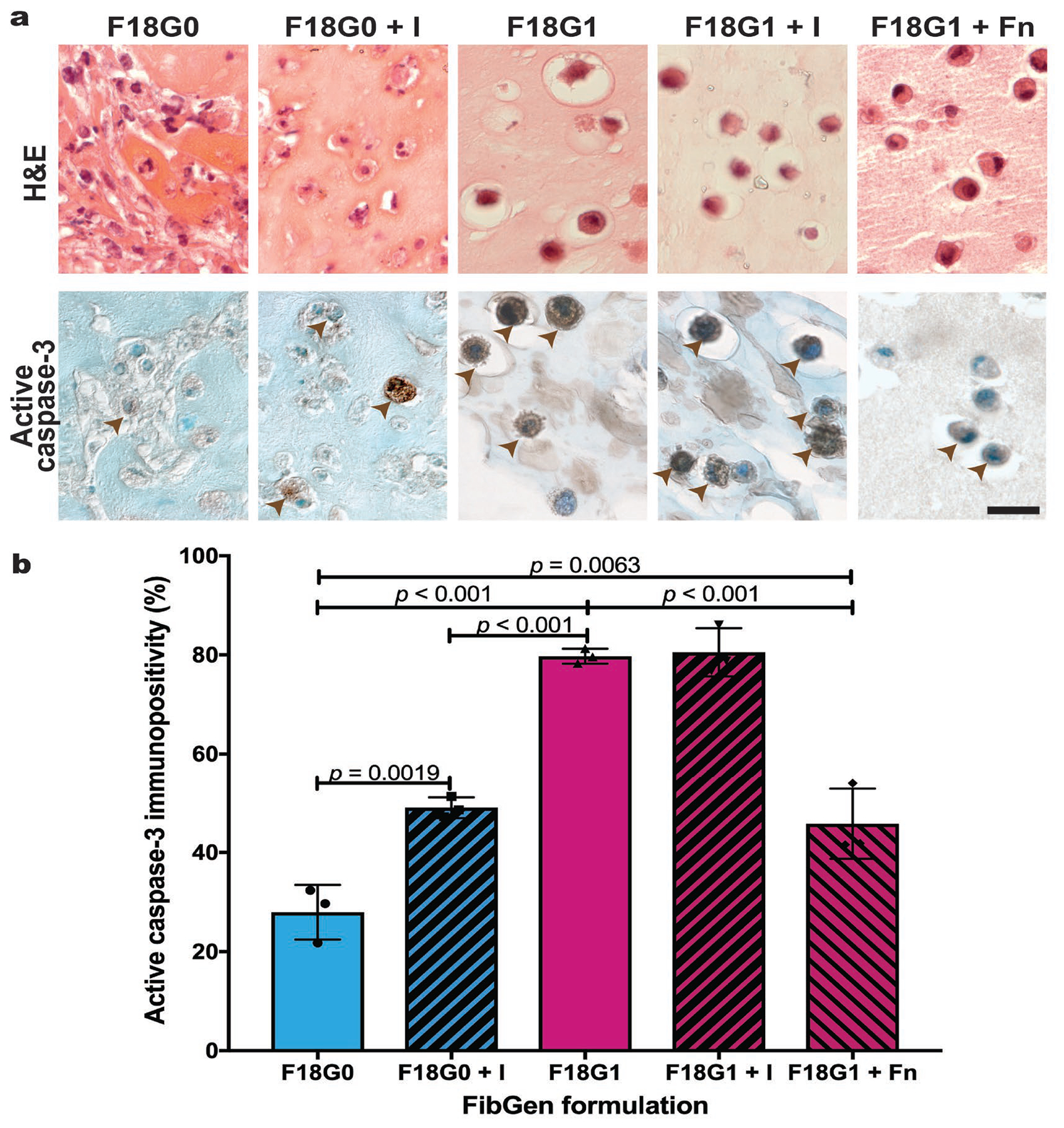
Genipin caused encapsulated AF cells to undergo apoptosis by preventing cell-matrix adhesions and cytotoxicity. (**a**) Day 5 H&E and active caspase-3 staining of FibGen formulations with decreased genipin concentration and modified cell-matrix binding potential. + I indicates culture treated with tirofiban hydrochloride, an inhibitor of the αIIbβ3 integrin recognition site on fibrin. Brown arrowheads indicate apoptotic cells. Scale bar: 20 μm. (**b**) Semiquantitative analysis of active caspase-3 positivity indicated significant differences based on genipin concentration, integrin inhibitor presence and fibronectin concentration. Data represented as mean ± standard deviation. *N* = 3 biological replicates; *n* = 3 technical replicates per biological replicate.

To determine the role of genipin on AF cell apoptosis levels, FibGen formulations with and without genipin were cultured with interventions that blocked or added AF cell integrin binding sites. A constant fibrin concentration of 18 mg/mL was chosen for all FibGen formulations in part 2b because similar density hydrogels have been shown to support cell viability without rapid degradation (Breen *et al.*, 2009). AF cells seeded in F18G0 (genipin-free) FibGen formulations displayed elongated processes, which are phenotypic of AF cells, and limited gap spaces between the encapsulated cells and hydrogel. Including genipin in the F18G1 FibGen formulation or culturing constructs in tirofiban hydrochloride (+ I) in the F18G0 + I formulation caused cells to become more rounded and display gap spaces between the FibGen matrix, indicating less effective biomaterial binding (Fig. 5a). Incorporation of fibronectin into in the F18G1 + Fn FibGen did not restore the elongated AF cell morphology but reduced the gap spaces between the cells and hydrogel. Semiquantitative analysis of active caspase-3 IHC (Fig. 5a) revealed that removing genipin significantly reduced apoptosis levels of encapsulated cells when compared with FibGen (F18G0 *vs*. F18G1, *p* < 0.001). Culturing constructs in the presence of the integrin inhibitor significantly increased apoptosis levels for fibrin (F18G0 *vs*. F18G0 + I, *p* = 0.0019) but showed no significant differences in genipin-containing conditions (F18G1 *vs*. F18G1 + I, *p* = 0.9996). Conversely, incorporating fibronectin into FibGen as a means of adding potential integrin binding sites significantly reduced apoptosis levels (F18G1 *vs*. F18G1 + Fn, *p* < 0.001). However, the recovery was only partial since fibronectin incorporation into FibGen could not reduce apoptosis levels to those of genipin-free conditions (F18G1 + Fn *vs*. F18G0, *p* = 0.0063) (Fig. 5b).

### Optimised formulation characterisation

**Fig. 6.**
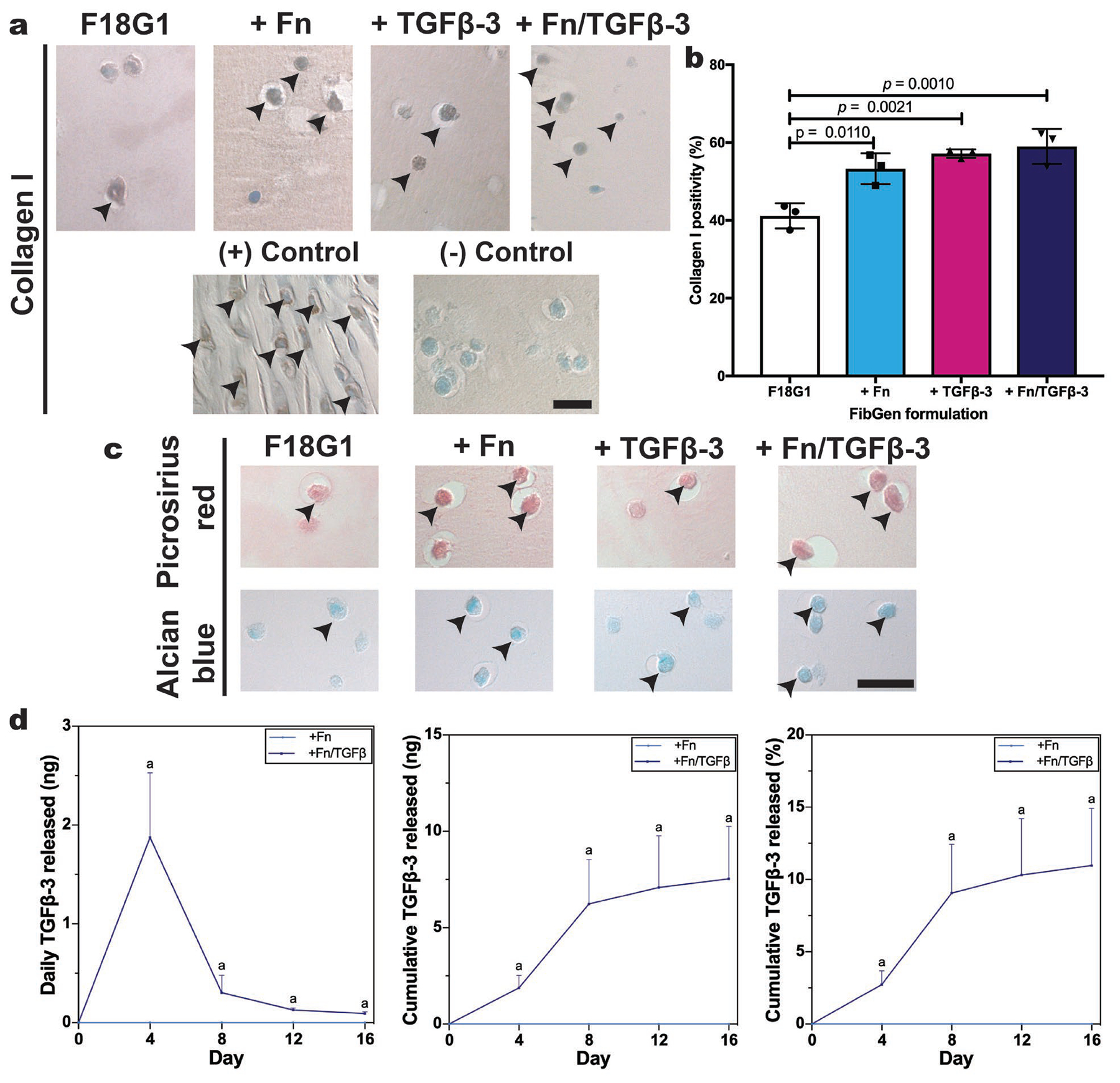
Fibronectin and TGFβ−3 supplementation enhanced ECM synthesis in optimised FibGen formulations. (**a**) Day 16 collagen I immunostaining of optimised low-modulus FibGen formulations. Black arrowheads indicate collagen I positive cells. Scale bar: 20 μm. (**b**) Semiquantitative analysis of collagen I positivity indicated significant increases in collagen I immunopositivity by adding fibronectin (+ Fn), TGFβ−3 (+ TGFβ−3) or both (+ Fn/TGFβ−3). Data represented as mean ± standard deviation. (**c**) Day 16 picrosirius red and alcian blue tinctorial stainings of optimised low-modulus FibGen formulations. Black arrowheads indicate positive staining for collagen content and proteoglycan/GAG deposition in the pericellular space. Scale bar: 20 μm. (**d**) ELISA analysis of medium TGFβ−3 concentration showed substantial and sustained release of TGFβ−3 throughout the 16-d culture period (^a^ *p* < 0.05). *N* = 3 biological replicates; *n* = 3 technical replicates per biological replicate.

Part 3 evaluated optimised low apoptosis FibGen formulations from part 2 in a 16 d culture experiment. ECM synthesis was evaluated with staining for collagen I and with picrosirius red and alcian blue. Semiquantitative analysis of collagen I IHC (Fig. 6a) showed that the addition of either fibronectin (+ Fn) or TGFβ−3 (+ TGFβ−3) enhanced collagen I immunopositivity. Adding both fibronectin and TGFβ−3 (+ Fn/TGFβ−3) showed the largest increase in collagen I immunopositivity (Fig. 6b). Qualitative assessment of picrosirius red and alcian blue tinctorial stainings showed a similar trend. Compared to the F18G1 formulation, adding fibronectin or TGFβ−3 resulted in a more robust staining; inclusion of both resulted in the largest increase in staining (Fig 6c). ELISA of medium used to culture TGFβ−3-loaded FibGen hydrogels showed that the + Fn/TGFβ−3 formulation was capable of sustained growth factor release. The medium concentration of TGFβ−3 was 26.4 ± 16.0 pM and 1.31 ± 0.44 pM at day 4 and 16, respectively. Cumulative growth factor release showed a steady increase over the 16 d culture period, resulting in 10.96 ± 6.86 % release of total loaded TGFβ−3 (Fig. 6d).

## Discussion

Cellular therapy using high-modulus biomaterial carriers offers promise to treat discogenic back pain because it can promote long-term repair while enabling immediate mechanical stabilisation. However, high-modulus materials designed to match biomechanical properties of the IVD are known to limit cell proliferation and drug release; in addition, there is a limited understanding of the factors limiting their biological performance (D’Este *et al.*, 2018). The study hypothesis was that FibGen formulations with lower macromer concentrations would enable more matrix synthesis of AF cells and growth factor release. The present three-part biomaterial study used AF cells in 3D cell culture systems with multiple FibGen formulations to identify critical biomaterial design criteria requiring further optimisation with three main findings. First, FibGen could release TGFβ−3 in a sustained manner at physiologically relevant levels. Second, the genipin crosslinking in FibGen prevented AF cells from accessing fibrin integrin binding sites, inducing anoikis and requiring the addition of fibronectin to reduce apoptosis. Third, ECM synthesis could be significantly improved by including additional integrin binding sites and supplementing with TGFβ−3.

Part 1 showed that dynamic culture conditions and TGFβ−3 supplementation could not increase AF cell ECM synthesis in a wide-range of high-modulus FibGen formulations. This result was somewhat surprising since TGFβ can direct mesenchymal stem cell differentiation toward IVD cell fate by upregulating ECM protein transcription *in vitro* (Risbud *et al.*, 2004; Stoyanov *et al.*, 2011) and TGFβ−3 supplementation increases AF cell ECM synthesis *in vitro* (Abbott *et al.*, 2013; Hondke *et al.*, 2018). AF cell ECM synthesis has been further improved *in vitro* using dynamic culture spinner flasks (Agrawal *et al.*, 2018) and shakers (Nerurkar *et al.*, 2011) to increase nutrient transport to cells. These prior studies have shown that TGFβ−3 and dynamic culture can stimulate ECM synthesis using less dense hydrogel scaffolds or more highly aligned electrospun scaffolds. Using collagen I IHC and tinctorial staining, the study findings showed that AF cells seeded in high-modulus FibGen formulations (F140G6, F70G6 and F70G1) lacked robust ECM synthesis in the presence of TGFβ−3 supplementation and dynamic culture. These results were comparable to previously published work with AF cells in high-modulus FibGen constructs (Cruz *et al.*, 2018). Overall, this indicates that these FibGen formulations, which are known to be biomechanically favourable, are not amenable to cell delivery. SEM imaging demonstrated that the limited cell viability could have been due to low porosity or poor cell matrix binding, which may have not allowed encapsulated cells to respond to exogenous cues. Previously reported studies investigating cell-seeded hydrogels showed that it is necessary to reduce macromer and crosslinker concentrations to promote significant matrix synthesis (Gupta and Nicoll, 2014; Lin *et al.*, 2016); thus, fibrin and genipin concentrations were varied in parts 2 and 3 of the study. Furthermore, since encapsulated AF cells synthesised minimal ECM, the potential of these high-modulus FibGen formulations for growth factor delivery was investigated.

FibGen formulations from part 1 released TGFβ−3, suggesting these high-modulus formulations could still be made to be bioactive. All tested FibGen formulations were capable of releasing TGFβ−3, with substantial drug release at culture day 2, known as burst release, and continued delivery at lower rates for 16 d. Hydrogels directly loaded with growth factors commonly experience burst release kinetics because release is controlled by diffusion and the largest concentration gradient exists early in culture (Censi *et al.*, 2012). Genipin crosslinker concentration was primarily responsible for controlling growth factor release from FibGen hydrogels bulk-loaded with TGFβ−3 since the F70G1 formulation released the largest amount and the F70G6 and F140G6 released at similar lower rates. The 6 mg/mL genipin formulations increased crosslinking, which decreased porosity and mesh size to significantly inhibit TGFβ−3 transport. Genipin crosslinking was previously shown to reduce average pore diameter and hydraulic permeability in loaded hydrogels (Dare *et al.*, 2009; Gamboa-Martínez *et al.*, 2015). Additionally, the genipin crosslinking reaction may sequester TGFβ−3 by crosslinking primary amine groups on the growth factor to fibrin (Butler *et al.*, 2003). The amount of TGFβ−3 released from the F70G1 FibGen formulation was at physiologically relevant concentrations, above ED50 (effective dose 50) values (Cheifetz *et al.*, 1990) over a 16 d culture period. FibGen formulations would also release larger amounts of TGFβ−3 with increased hydrogel degradation *in vivo* due to the presence of plasmin (Likhitpanichkul *et al.*, 2014). Therefore, it is reasonable to conclude that F70G1 formulations could be used for growth factor delivery for sustained durations and this formulation was previously shown to hold biomechanical properties that approach AF tissue levels and enhance motion segment properties (Cruz *et al.*, 2018).

Part 2a evaluated if reduced fibrin macromer concentration would enable FibGen formulations to be more amenable for cell delivery and determined that varying fibrin macromer concentration had little effect on AF cells matrix elaboration or apoptosis levels. Hydrogel macromer concentration impacts the porosity and stiffness of the resultant constructs (Li *et al.*, 2018); thus, varying fibrin macromer concentration varied the porosity and stiffness of FibGen constructs. Reducing fibrin macromer concentration in FibGen to 5 mg/mL did not reduce apoptosis-marker positivity below 75 % while genipin remained at 1 mg/mL concentration (Fig. 4). Since no significant differences in apoptosis levels were found based on macromer concentration, it was concluded that FibGen macromer density, porosity and stiffness were not the primary drivers of high apoptosis levels. Previous work seeding AF cells in FibGen showed high viability (Cruz *et al.*, 2018); thus, appropriate staining controls and multiple biological replicates for two independent apoptosis measurement methods were used to validate the study findings. The discrepancy between the present and a prior study (Cruz *et al.*, 2018) was attributed to differences in staining methods since calcein can still stain live cells undergoing apoptosis. Assessment of the degree of apoptosis was used in place of measurement of ECM synthesis in part 2 because it was considered an essential estimation of cell health. Furthermore, fibrin scaffolds with similar macromer concentrations without genipin exhibited ECM synthesis without high levels of apoptosis (Bensaïd *et al.*, 2003; Wang *et al.*, 2018). Consequently, hydrogel macromer concentration was not considered to be a critical factor in causing apoptosis.

Part 2b determined that genipin crosslinking was a cause of apoptosis for cell-seeded FibGen and confirmed that removing genipin in the F18G0 formulation significantly reduced apoptosis levels. AF cells encapsulated in F18G0 formulations took on an elongated morphology indicative of enhanced cell-matrix binding (Fig. 5). Cell-matrix interactions are important for a variety of integrin-mediated signalling (Giancotti and Ruoslahti, 1999); thus, facilitating interactions between encapsulated cells and the biomaterial carrier is a critical design requirement for tissue engineering strategies (Parisi *et al.*, 2018). Disruption of cell-matrix contacts in anchorage-dependent cells induces apoptosis and this phenomenon is termed anoikis (Frisch and Ruoslahti, 1997). AF cell-matrix interactions were identified to be critical for preventing apoptosis because inhibiting cell-matrix interactions in fibrin hydrogels by using the tirofiban hydrochloride inhibitor in the F18G0 (F18G0 + I) formulation caused encapsulated cells to take on a rounded morphology and undergo apoptosis. Genipin crosslinking caused a similar rounded morphology and increased apoptosis to even higher levels. Since FibGen with tirofiban hydrochloride (F18G1 + I) did not increase apoptosis more than FibGen alone (F18G1), this suggested that genipin crosslinking inhibited AF cell-matrix interactions in FibGen constructs. As a result, an additional ‘rescue’ experiment was performed where integrin binding sites were added by incorporating fibronectin into FibGen.

Incorporating fibronectin into FibGen partially rescued AF cell apoptosis (F18G1 + Fn *vs*. F18G0), suggesting that inhibition of the interactions between AF cells and the FibGen matrix due to genipin crosslinking is an important contributor to the large amount of apoptosis observed in FibGen. However, anoikis only explained a portion of the apoptosis observed since the percentage increase in apoptosis levels from culturing genipin-free FibGen formulations with a specific integrin inhibitor (F18G0 *vs*. F18G0 + I) was approximately equal to the percentage decrease in apoptosis levels when encapsulating cells in FibGen formulations that included fibronectin to increase potential integrin bindings sites (F18G1 *vs*. F18G1 + Fn) (Fig. 5b). The remaining apoptosis levels, which could not be modified by cell-matrix interactions, indicated that genipin crosslinking had additional cytotoxic effects. Compared to other crosslinkers, genipin is considered to have low cytotoxicity (Oryan *et al.*, 2018) but direct cytotoxic effects of genipin on IVD cells have been recently reported *in vitro* (Frauchiger *et al.*, 2018). Importantly, genipin cytotoxicity effects are known to be acute because AF cells can proliferate on the surface of acellular FibGen following gelation, with potential for cell migration into the gel, further highlighting that acellular FibGen can be used as a bioactive AF sealant (Guterl *et al.*, 2014; Likhitpanichkul *et al.*, 2014).

Part 3 demonstrated that the incorporation of fibronectin or TGFβ−3 into low-modulus FibGen constructs enhanced AF cell ECM synthesis, with incorporation of both showing the largest increase. Similarly, low-modulus methylcellulose biomaterials for NP repair show lower macromer concentrations and promote more ECM synthesis (Gupta and Nicoll, 2014). Results build on this finding by characterising how cell-matrix interactions and growth factor supplementation impacted ECM synthesis using a different low-modulus biomaterial. Inclusion of fibronectin (+ Fn) or TGFβ−3 (+ TGFβ−3) into the low-modulus F18G1 formulation significantly increased ECM synthesis. ECM synthesis was augmented when both fibronectin and TGFβ−3 were included into FibGen hydrogels (+ Fn/TGFβ−3). This sort of sequential optimisation of cell-matrix interactions and growth factor supplementation is important for the classical tissue engineering framework (Langer and Vacanti, 1993) and the present study highlighted the need to prioritise cell-biomaterial binding in cell delivery strategies. Furthermore, these discoveries in IVD tissue engineering supported a similar study performed in tendon tissue engineering by Chang *et al.* (2020) who modified their biomaterial to enhance cell-biomaterial interactions before experimenting with growth factor supplementation. In addition to promoting delivered AF cells to synthesise ECM, the F18G1 + Fn/TGFβ−3 FibGen formulation released TGFβ−3 at physiologically relevant levels throughout the culture period, which could enhance native IVD cell ECM synthesis. Therefore, it is possible to conclude that this optimised construct could effectively deliver biological factors to the injured IVD, which may enhance endogenous repair, while also delivering viable cells capable of ECM synthesis for exogenous repair.

The study used *in vitro* cell culture models because a high degree of control was required to identify specific critical factors important in limiting ECM synthesis and causing apoptosis of encapsulated AF cells. Bovine AF cells are commonly used and the specific AF cell species is not expected to impact upon the conclusions. Part 1 used only a single biological replicate, yet it is the authors’ opinion that results were conclusive since limited ECM synthesis was confirmed across all formulations with multiple technical replicates and because part 2 studies confirmed high apoptosis levels with 3 biological replicates and 3 technical replicates per animal. The study highlighted the critical need for high-modulus cell carriers to be engineered to have sufficient numbers of integrin binding sites and the capacity to protect cells against acute crosslinker cytotoxicity. The study directs future refinements of these bioactive formulations to better reduce the challenges of genipin crosslinking prior to eventual *in vivo* testing, perhaps using cellular encapsulation methods as previously described (Gasperini *et al.*, 2014).

## Conclusions

High-modulus FibGen formulations with low crosslinker concentration may be suitable for delivering biologically active compounds since TGFβ−3 was delivered at effective doses for sustained durations while also enabling some mechanical stabilisation. Genipin, and not fibrin macromer, concentration was the critical component of FibGen that caused anoikis by preventing AF cells from accessing integrin binding sites. Fibronectin increased integrin binding sites and prevented a portion of the encapsulated cell apoptosis, allowing encapsulated cells to increase ECM synthesis. Therefore, engineering the cellular microenvironment with appropriate signalling molecules is crucial for developing AF repair biomaterials that are mechanically competent and cytocompatible. Additionally, cell-encapsulation strategies must be further developed to protect AF cells against acute crosslinker cytotoxicity and further enhance high-modulus FibGen for AF cell delivery.

## Discussion with Reviewer

Zhen Li: Are there some other methods to enhance integrin binding sites other than incorporation of fibronectin?

Authors: Integrin binding sites can be increased by incorporating RGD sequences, which are present in fibronectin, directly onto fibrin. Other families of integrin binding motifs are also available; however, the RGD is the most common.

Zhen Li: A high concentration of genipin crosslinking results in high mechanical property of fibrin-genipin glue but poor cytocompatibility. A low concentration of genipin crosslinking would improve the cytocompatibility of the gel but worsen the mechanical properties. Would a bilayer design facilitate the AF repair requirement, *e.g*. with a biocompatible layer inside to enhance interface healing and an adhesive layer outside to close the AF defect?

Authors: A bilayer design would be an interesting approach to achieve the desired biological and biomechanical properties; however, this would make translation a bit more difficult and likely require 2 injections. Our future biomaterial design goals are focused on achieving these two objectives with an injectable biomaterial that can protect encapsulated cells in the short term, then provide space in the material or proliferation and ECM synthesis in the long term.

## List of Abbreviations

3D: three-dimensional
AF: annulus fibrosus
ANOVA: analysis of variance
DAPI: 4′,6-diamidino-2-phenylindole
DI: deionized
DMEM: Dulbecco’s modified Eagle’s medium
DMSO: dimethyl sulphoxide
ECM: extracellular matrix
ELISA: enzyme-linked immunosorbent assay
FBS: foetal bovine serum
FibGen: genipin-crosslinked fibrin
GAG: glycosaminoglycan
H&E: haematoxylin and eosin
HRP: horseradish peroxidase
IHC: immunohistochemistry
IVD: intervertebral disc
NP: nucleus pulposus
PBS: phosphate-buffered saline
PS: penicillin-streptomycin
SEM: scanning electron microscopy
TGFβ−3: transforming growth factor beta-3
TUNEL: terminal deoxynucleotidyl transferase dUTP nick end labelling
